# A novel food-based negative oral contrast agent compared with two conventional oral contrast agents in abdominal CT: a three-arm parallel blinded randomised controlled single-centre trial

**DOI:** 10.1186/s41747-022-00267-z

**Published:** 2022-04-05

**Authors:** Peter Leander, Georgios Stathis, Lucia Casal-Dujat, Karolina Boman, Ingvar Adnerhill, Jan Marsal, Olof Böök, Thomas Fork

**Affiliations:** 1 Peritus Clinic AB, Scheelevägen 8, 223 63 Lund, Sweden; 2 Department of Translational Medicine, Skåne University Hospital, Lund University, 205 02 Malmö, Sweden; 3 Lument AB, Scheelevägen 22, 223 63 Lund, Sweden; 4 Department of Oncology, Skåne University Hospital, 205 02 Malmö, Sweden; 5 Department of Gastroenterology, Skåne University Hospital, 205 02 Malmö, Sweden; 6 Department of Clinical Sciences Lund, Lund University, 221 84 Lund, Sweden

**Keywords:** Contrast media, Iohexol, Intestine (small), MoviPrep, Tomography (x-ray computed)

## Abstract

**Background:**

A negative oral contrast agent (OCA) has been long sought for, to better delineate the bowel and visualise surrounding structures. Lumentin® 44 (L44) is a new OCA formulated to fill the entire small bowel. The aim of this study was to compare L44 with positive and neutral conventional OCA in abdominal computed tomography (CT).

**Methods:**

Forty-five oncologic patients were randomised to receive either L44 or one of the two comparators (MoviPrep® or diluted Omnipaque®). Abdominal CT examinations with intravenous contrast agent were acquired according to standard protocols. The studies were read independently by two senior radiologists.

**Results:**

The mean intraluminal Hounsfield units (HU)-values of regions-of-interest (ROIs) for each subsegment of small bowel and treatment group were -404.0 HU for L44, 166.1 HU for Omnipaque®, and 16.7 HU for MoviPrep® (L44 *versus* Omnipaque, *p* < 0.001: L44 *versus* MoviPrep *p* < 0.001; Omnipaque *versus* MoviPrep, *p* = 0.003). Adverse events, only mild, using L44 were numerically fewer than for using conventional oral contrast agents. Visualisation of abdominal structures beyond the small bowel was similar to the comparators.

**Conclusions:**

L44 is a negative OCA with luminal radiodensity at approximately -400 HU creating a unique small bowel appearance on CT scans. The high bowel wall-to-lumen contrast may enable improved visualisation in a range of pathologic conditions. L44 showed a good safety profile and was well accepted by patients studied.

**Trial registration:**

EudraCT (2017-002368-42) and in ClinicalTrials.gov (NCT03326518).

**Supplementary Information:**

The online version contains supplementary material available at 10.1186/s41747-022-00267-z.

## Key points

- Lumentin 44 (L44) is a negative oral contrast agent for computed tomography.
- L44 gave excellent bowel wall-to-lumen contrast that may enable better diagnostics.
- L44 was well tolerated by the studied patients.

## Background

Abdominal computed tomography (CT) is one of the most common examinations performed in abdominal radiology. Many protocols exist how to perform the examinations most often including as well intravenous iodinated contrast to enhance certain structures as well as oral contrast agent (OCA) to delineate the small bowel (SB) [1].

Some authors today advocate to perform abdominal CT without any OCA [2, 3], while other advocate its use especially in non-acute patients [4]. Many discussions concerning the use of or not use of OCA refers to acute examinations. Exact figures of frequency of use of OCA are hard to find in the literature and have changed to be less frequent during later years. In more aimed diagnostics of the SB, experience from advanced therapeutic endoscopy and video capsule enteroscopy has shown that there is still a place for radiological imaging of the whole SB [5, 7]. If so, it is the belief of many that the use of OCA prior to abdominal CT will facilitate a correct diagnosis.

When iodinated OCA are used to fill the SB, the luminal material shows high density values measured in Hounsfield units (HU), providing a so-called positive oral contrast. The filling of the SB in this case is close to that of the SB wall and may thereby conceal small details of the wall and decrease the ability to depict wall lesions as thickened and inflamed bowel wall.

To overcome the limitations of positive oral contrast, water or water containing inert sugars to retain water in the lumen of the SB has been used. These media give luminal density values in the range from 0 to 20 HU and are thus denominated neutral oral contrast. Protocols using neutral oral contrast are often used for detection of pathology in the stomach and small pancreatic head tumours and in CT-enterography examinations [7–9]. However, a neutral oral contrast may in some instances be less favourable when cystic lesions are present, especially in the pelvis. In this case, the luminal attenuation of the SB will have the same or close to the same attenuation as the pathologic lesions to be detected. In addition, water is rapidly absorbed in the SB and thereby may limit its performance in demarcating the distal SB.

As outlined above, there are limitations with both positive and neutral OCAs. Therefore, radiologists have for decades expressed a need for lower attenuation in the SB compared to surrounding structures, *i.e.*, negative oral contrast, to improve diagnostic capabilities [10–14]. Many of these studies have used fat-containing material. For the present study, a fat-free material with lower attenuation than fat has been invented [15]. This newly developed contrast medium, named Lumentin® 44 (L44), is food-based and displays HU-negative attenuation. Importantly, the attenuation in the SB using L44 is outside the range of any physiological structure in the body as well as outside that of air. L44 is thus physiologically “HU-unique” in its attenuation. The first series of L44 abdominal CT examinations on healthy, adult volunteers were highly encouraging with enhanced bowel wall-to-lumen contrast, good patient acceptance, and safety profile [15].

The L44 content of air (44%) in water may explain the low frequency of adverse events (AEs), seen in the first study, foremost lack of diarrhoea. Positive oral regimes, especially if inert sugars as sorbitol are added with its’ osmotic pressures, are known to be poorly tolerated by many patients. A cautious attitude is required in patients with ongoing oncologic therapy that may affect the SB, to avoid aggravation of adverse symptoms.

In the present study, L44 was tested in ill subjects receiving oncologic treatment. L44 was compared with two commonly used regimes of OCAs, *i.e.*, diluted Omnipaque® [9] and MoviPrep® [16].

The primary aim of the study was to calculate bowel wall-to-lumen attenuation differences. Secondary aims were to assess (i) the distribution of L44 in the abdomen, (ii) signs of degradation of L44, and (c) safety and subjective patients’ impression of L44.

## Methods

The trial was conducted in compliance with national and international standards including the European Union Data Protection Directive (95/46/EC) and the Declaration of Helsinki. The institutional review board approved the study (2015/912), and each patient gave written consent for their participation. Study design was an open, randomised, controlled, single centre trial in to evaluate image quality and tolerability of Lumentin® 44 in abdominal CT, in comparison with diluted Omnipaque and MoviPrep.

**Fig. 1:**
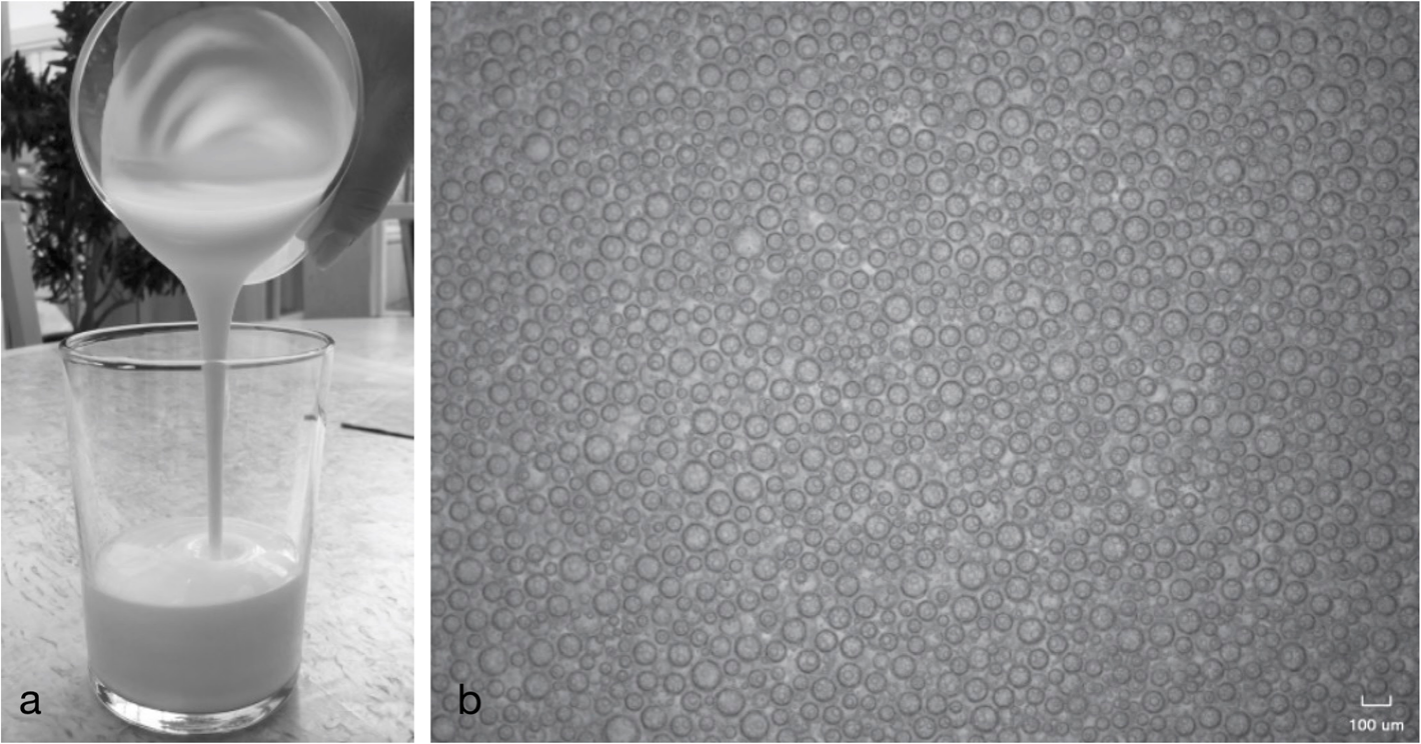
**a** Freshly prepared Lumentin 44 (L44) poured into a glass. **b** Optical microscopy image of L44, ready to be administered, which consists of spherical air micro-bubbles dispersed in an aqueous phase that contains protein as foaming agent, stabiliser, buffer agent, and flavouring

The OCA (L44) has been described in an earlier publication [15]. In short, L44 is a liquid foam, with 44% air content, made up of air micro-bubbles dispersed in a continuous aqueous phase, containing protein, stabiliser, buffer agent, and flavouring (Fig. 1a, b). *In vivo* administration of L44 had no effects on serum electrolytes [15]. The foam was freshly prepared on site and approved for administration after controlling the homogeneity of the foam (ocular inspection of the jar containing the foam while having it tilted and illuminated). Foam with phase separation (liquid drainage) and/or visible distinguishable bubbles were discarded.

Patients were recruited from the Department of Oncology for suspected tumour, staging, or follow-up between November 2017 and February 2019, Additional file 1.

Taking the difference in wall to lumen contrast density in consideration (78% power, 2-sided *p* < 0.050), the sample size calculation was: 40 patients for L44, 20 patients for Omnipaque, and 20 patients for MoviPrep. A prerequisite to achieve difference in wall to lumen contrast density is distribution of the OCA throughout the SB. As this distribution was not known on beforehand, it was taken in consideration as an uncertain factor and thereby the need for more patients when sample size was calculated.

Subjects aged 18 and older were randomised to the three arms. However, an interim analysis, performed at midterm, showed that the primary endpoint had been reached with high statistical significance. As a consequence, the series was stopped after 45 patients had been recruited.

With experience from a previous study [15], the recommended dose for L44 was 1,000 ± 200 mL. Subjects having ingested less than 750 mL were withdrawn from the trial. MoviPrep and diluted Omnipaque were administered according to the standard protocol of the Department of Radiology: 30 mL of Omnipaque 240 mg I/mL and 30 mL 70% sorbitol dissolved in 1,000 mL of water; MoviPrep, units A and B, dissolved in 1,000 mL of water. A steady intake over 45 to 60 min was recommended for all three oral regimes.

The CT examination commenced within 10 min after finishing the intake of OCA. Examinations were performed on a Siemens Somatom Edge (Siemens Healthineers, Forchheim Germany). A standard clinical abdominal protocol with intravenous administration of an iodinated contrast agent was administered at a dose of 500 mg I/kg body weight. Bolus triggering technique in aorta and post-trigger delay of 50 s were used (routine imaging in the portal venous phase). Technical parameters were 120 kVp, 250 reference mAs, reconstruction filter, *i.e.*, kernel B30f, standard pitch, iterative reconstruction, axial, coronal and sagittal series reconstructed and archived in the picture archiving and communication system (PACS).

Two experienced abdominal radiologists, with 20 and 5 years of experience, read all examination independently, without knowledge of the patients’ history.

The primary endpoint was defined as the obtained, agent-dependent, bowel wall-to-lumen contrast. For this purpose, two 6-mm luminal regions of interest (ROI) were drawn in the duodenum, jejunum, proximal ileum, and distal ileum and one ROI in the terminal ileum, nine in total per patient. The standard department PACS using circular ROIs were used for the measurements and mean and standard deviation within these regions were obtained and filed electronically. After repetitive measurements, a mean representation of the wall density was set to 80 HU for all images. Especially with Omnipaque, it was not possible to discriminate the thin wall when the bowel was filled, as both are depicted white on images. The difference in attenuation between bowel wall and lumen was calculated by subtracting the mean HU in ROIs with the chosen HU value 80 of the wall.

Secondary endpoints comprised bowel filling properties, *i.e.*, extension and distension of the three agents. Results were graded according to a scale from 1 to 9 [17, 18] (Table 1). Another secondary endpoint was to evaluate the ability to delineate: (a) the SB; (b) parenchymal organs; and (c) mesentery including lymph nodes, vessels, and omentum. For these estimations, results were graded according to a scale from 1 to 9 (1 = low diagnostic capability; 9 = high diagnostic capability). Secondary variables also included assessment of foam degradation of L44, *i.e.*, formation of distinguishable large bubbles and signs of phase separation, giving a foam-fluid level seen on the CT-images. All patients assessed taste, smell, consistency, ability to swallow, and fullness after swallowing the oral contrast, and their impressions were noted according to a 5-grade ordinal scale, where the highest value was the most favourable. Safety data set included all randomised subjects who drank at least some of the contrast agent.

**Table 1:** Likert scale grading of oral contrast distribution in the small bowel (extension and distension)

Extension	Grade	Distention
Segment filled to 100%	9	Excellent or almost over distended
Filling > 75% but < 100%	8	Optimal filling
Filling about 75%	7	Good filling
Filling > 50% but < 75%	6	Slightly better than grade 5
Filling about 50%	5	Medium filled bowel loop
Filling > 25% but < 50%	4	Amount of OCA just allowing for a ROI of 6 mm
Filling about 25%	3	Small amount of OCA, insufficient for placing a ROI of 6 mm
Traces of OCA filling	2	Minimal amount of OCA
No OCA identified	1	No OCA identified

*OCA* Oral contrast agent; *ROI* Region of interest

To compare the results in the treatment groups for continuous and ordinal variables, the Wilcoxon rank sum test was applied. To compare binary variables between the groups, the Fisher exact test was applied. In each of the tests, a 2-sided *p*-value lower than 0.05 was considered significant.

## Results

In total, 45 patients were randomised, 19 to L44, 12 to Omnipaque, and 14 to MoviPrep. Thirty-five patients were under treatment for current malignancies and 10 were on maintenance therapy. Two patients who had ingested an insufficient volume of L44, and two others who withdrew their consent, were excluded. Forty-one subjects completed the study and were included in the statistical analyses: 15 in the L44-group, 12 in the Omnipaque-group, and 14 in the MoviPrep-group. Out of 44 safety sets, 43 were qualified for full analysis. Demographic details, *i.e.*, distribution of sex and age groups, revealed nothing untoward. The mean age was 63.0 years (range 20 to 86). The volume of L44 consumed was significantly lower compared to the amount of Omnipaque as was the volume of L44 to body mass index (Table 2), Additional file 2.

**Table 2:** Ingested contrast volume in relation to patient characteristics in the three study arms

Group		Body mass index	Oral contrast agent (mL)	Oral contrast agent/body mass index
Lumentin 44 (L44) (*n* = 17)	Mean ± SD	29.9 ± 5.9	997.5 ± 43.7	34.4 ± 6.0
Omnipaque (*n* = 12)	Mean ± SD	25.4 ± 3.6	1,061 ± 34.6	42.4 ± 5.5
MoviPrep ± *n* = 14)	Mean ± SD	25.2 ± 3.5	988.64 ± 150.8	39.6 ± 6.2
*p*-values^a^	L44 *versus* Omnipaque	0.021	< 0.001	0.002
	L44 *versus* Moviprep	0.012	0.474	0.017

*SD* Standard deviation

^a^Wilcoxon rank sum test (2-sided)

**Fig. 2:**
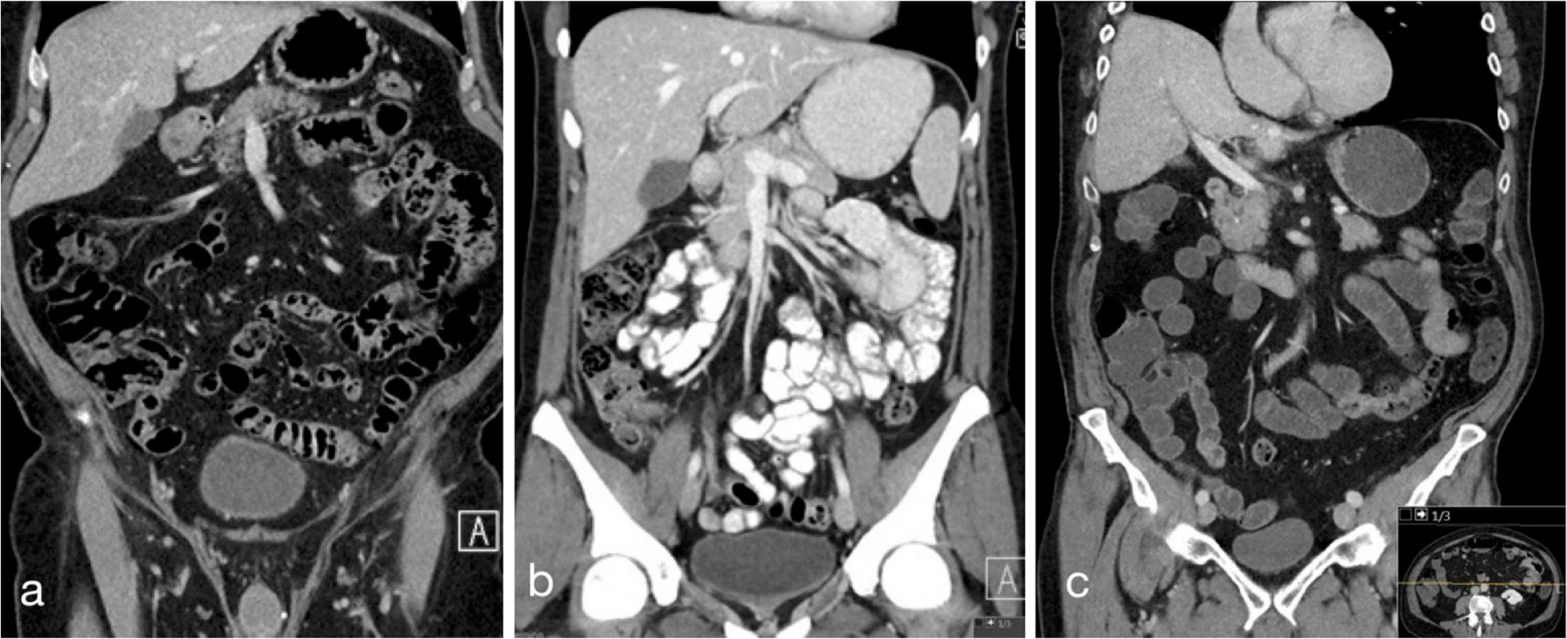
Abdominal computed tomography coronal view after oral administration of Lumentin 44 (**a**), diluted Omnipaque (**b**), and MoviPrep (**c**). Standard abdominal window-setting (window width 400 HU, window level 60 HU)

**Fig. 3:**
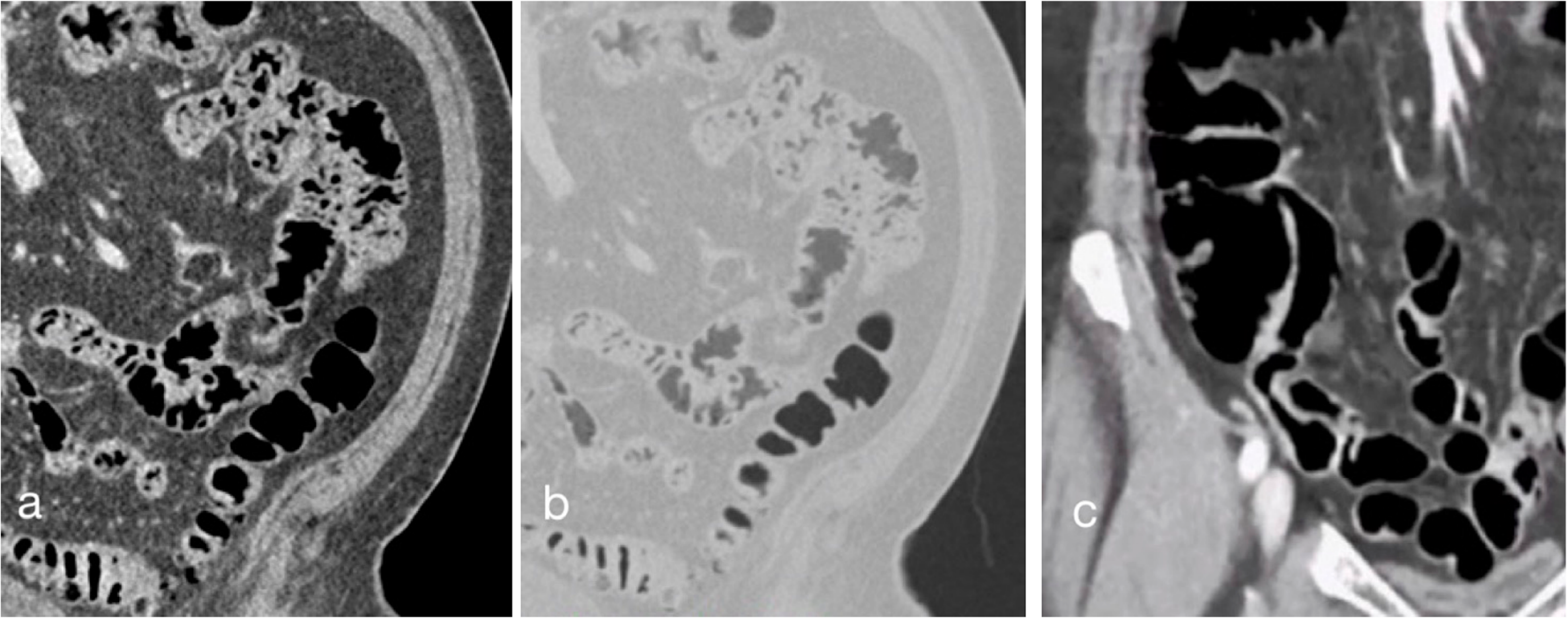
Lumentin 44 in small bowel (SB) loops. Close up coronal views showing subtle details in SB walls. **a** Abdominal window setting (WW 400 HU, WL -15 HU) and 0.7 mm thin-slice reconstruction showing mucosal folds. **b** Lung window setting (WW 2,000 HU, WL -400) and 0.7 mm thin-slice reconstruction showing SB filled with Lumentin 44 and part of sigmoid colon containing air. **c** Abdominal window setting (WW 400 HU, WL -15 HU) and 5 mm thick-slice reconstruction showing terminal ileum entering the caecum. WW, Window width; WL, Window level

Typical abdominal CT-images with L44, Omnipaque, and MoviPrep as bowel filling agents are shown in Figs. 2 and 3. Additional cine images are available as Additional files 3, 4 and 5.

The mean intraluminal HU-values of ROIs for each subsegment of SB and treatment group were -404.0 HU for L44, 166.1 HU for Omnipaque, and 16.7 HU MoviPrep. The mean difference between the contrast filled bowel lumen and iodine-enhanced bowel wall (the second one set to 80 HU) was significantly higher for L44 in all segments compared to comparators. The differences between L44 and the other two groups, respectively, were highly significant (Table 3).

**Table 3:** Mean differences in HU between contrast filled bowel lumen and iodine-enhanced bowel wall (set to 80 HU as a standard)

Variable	Lumentin 44 (*n* = 17)	Omnipaque (*n* = 12)	MoviPrep (*n* = 14)	*p*-values^a^		
				L44 *versus* Omnipaque	L44 *versus* MoviPrep	Omnipaque *versus* MoviPrep
Duodenum ROI 1	486.3 (210.8	147.0 (291.3	48.1 (18.7	0.001	< 0.001	0.076
Duodenum ROI 2	414.6 ± 239.0	133.0 ± 293.4	43.0 ± 19.0	< 0.001	< 0.001	0.699
Jejunum ROI 1	490.3 ± 176.0	115.0 ± 88.6	57.5 ± 8.7	< 0.001	< 0.001	0.015
Jejunum ROI 2	496.0 ± 171.5	130.0 ± 104.6	61.6 ± 10.9	< 0.001	< 0.001	0.007
Proximal ileum ROI 1	582.0 ± 201.0	150.4 ± 120.5	66.9 ± 4.1	< 0.001	< 0.001	< 0.001
Proximal ileum ROI 2	580.0 ± 208.0	151.9 ± 124.6	67.3 ± 3.9	< 0.001	< 0.001	0.007
Distal ileum ROI 1	477.1 ± 313.3	118.8 ± 144.4	65.4 ± 4.7	< 0.004	0.002	> 0.999
Distal ileum ROI 2	398.8 ± 302.7	117.2 ± 130.6	64.2 ± 5.3	0.018	< 0.004	0.368
Terminal ileum, ROI	396.6 ± 326.6	66.9 ± 41.0	99.1 ± 131.2	0.018	0.007	0.898
Mean difference for all sites	484.0 ± 192.4	122.1 ± 81.4	64.5 ± 15.9	< 0.001	< 0.001	0.003

Data are given as mean ± standard deviation. *HU* Hounsfield units, *ROI* Region of Interest

^a^Wilcoxon rank sum test (2-sided)

**Fig. 4:**
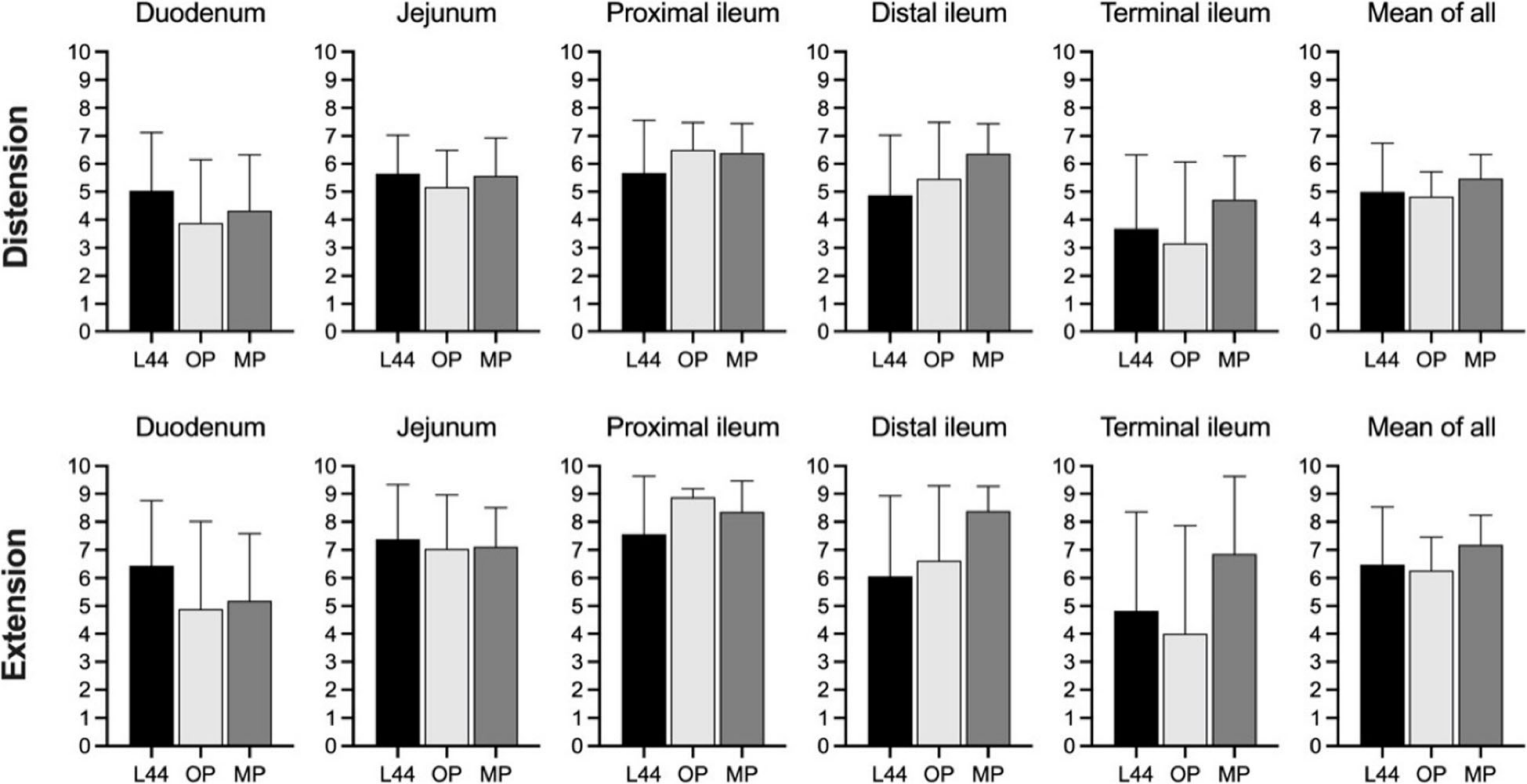
Measurements of oral contrast agent (OCA) distribution. Distension: mean ± standard deviation grade of distension by OCA in subsegments of the small bowel. Extension: mean ± standard deviation grade of extension in subsegments of the small bowel by OCA. Grading 1–9 according to Table 1. Number of patients: L44 (*n* = 17); diluted OP (*n* = 12); MP (*n* = 14). L44, Lumentin 44; OP, Omnipaque; MP, MoviPrep

L44 showed good and consistent bowel filling properties (Table 1), *i.e.*, distension  and extension (Fig. 4) and did not differ between SB segments nor when taking the whole SB into account (L44 *versus* Omnipaque for distension, *p* = 1.000, for extension, *p* = 0.723; L44 *versus* MoviPrep for distension, *p* = 0.321, for extension, *p* = 0.462). The effect of each OCA on the radiologists image reading (analysed for SB, parenchymal organs and mesentery including lymph nodes, vessels and omentum) showed means (standard deviation) 7.18 (0.79) for L44, 6.65 (0.99) for Omnipaque, and 6.89 (0.73) for MoviPrep. There were no statistical differences between L44 and Omnipaque (*p* = 0.176), nor between L44 and MoviPrep (*p* = 0.328). Degradation of L44 was overall low with a single sign of presence of detectable large bubble in one patient and phase separation in three patients shown in the CT images.

Patients’ quality assessments expressed in median grades of taste and smell were 4 for both L44 and Omnipaque and 3 for MoviPrep (L44 *versus* MoviPrep 0.006 and Omnipaque *versus* MoviPrep 0.0027). The median grade of easiness to swallow was 3 for L44 and MoviPrep and 5 for Omnipaque (*p* < 0.001), and the median grade for sensation of fullness (lower meaning better) was 2 for L44 and 3 for both Omnipaque (*p* = 0.001) and MoviPrep (*p* = 0.07).

The safety data set included 18 of 19 subjects in the L44 group (94.7%) and all subjects in the Omnipaque and MoviPrep groups. No serious AEs or suspected unexpected serious adverse reactions were recorded during the trial, and no subjects withdrew from treatment or had the dose interrupted due to AEs. No severe AEs were reported. The various AEs are shown in detail in Table 4. The type and frequencies of AEs were similar between groups, except for diarrhoea which was reported by nearly half of the patients in the MoviPrep group, by 25% in the Omnipaque group, but by none of the patients in the L44 group.

**Table 4:** Adverse events by treatment groups

Type of adverse event	Lumentin 44 (*n* = 18)		Omnipaque (*n* = 12)		MoviPrep (*n* = 14)	
	Adverse events (*n*)	Patients, *n* (%)	Adverse events (*n*)	Patients, *n* (%)	Adverse events (*n*)	Patients, *n* (%)
Gastrointestinal disorders	10	7 (38.9)	13	7 (58.3)	14	8 (57.1)
Abdominal distension	1	1 (5.6)	1	1 (8.3)	1	1 (7.1)
Epigastralgia	1	1 (5.6)	3	3 (25.0)	3	3 (21.4)
Diarrhoea	0	0 (0.0)	3	3 (25.0)	6	6 (42.9)
Eructation	1	1 (5.6)	0	0 (0.0)	0	
Flatulence	4	4 (22.2)	4	4 (33.3)	3	3 (21.4)
Nausea	2	2 (11.1)	2	2 (16.7)	1	1 (7.1)
Rectal haemorrhage	1	1 (5.6)	0	0 (0.0)	0	0 (0.0)
General disorder, chills	0	0 (0.0)	1	1 (8.3)	0	0 (0.0)

## Discussion

The current investigation shows that L44 gives a high wall-to-lumen contrast and distributes well in SB. As such, L44 may be a better alternative than positive or neutral oral contrast. However, so far, L44 has only been studied from a technical point of view, and future studies must be designed to see if technical advantages generate improved and more confident diagnostic performance.

To use L44 and its properties as a new type of oral contrast may be a challenge for radiologist as they are not accustomed to see dark SB lumen on CT-images. However, if needed, L44 is easily differentiated from air on images by choosing a lung window setting at the reading station thereby turning L44 grey whereas air stays black. In addition, radiologists are familiar with T1-weighted MRI-images of the SB with “black” SB loops, *i.e.*, images with many similarities with those of CT with L44.

The ability to perform diagnostics on abdominal CT with “black” bowels was also preliminary tested with some few parameters in the present investigation and shown to be uninfluenced by L44 compared to the other two regimes.

L44 is a food based micro-foam in which air is incorporated in an aqueous dispersion, generating a homogenous drinkable liquid foam with reproducible and consistent characteristics of air content, bubble size, homogeneity, consistency, stability, and palatability. Properly prepared, the foam is stable for hours, keeping its properties required for the intended imaging purpose unaffected during its oral administration, passage through the stomach, and distribution along the intestinal lumen. Foamability and foam life depend on its composition, preparation method, and foam degradation mechanism [19, 20]. The air trapped in the foam is the key ingredient responsible for the SB appearance with negative HU-values between those of fat and air, as well as the improved demarcation of the luminal interface of the SB surface. Foam degradation in terms of visible large bubbles and phase separation were only seen by one of the investigators in single cases. Retained endogenous bowel fluids as a cause for the observed foam-fluid level could not be excluded.

Other oral contrast regimens are easy to dispense, and handling L44 requires a preparation. To not increase work burden in departments, there is an ongoing development of an automatic dispenser for the product to be tested for upcoming studies.

The lightness of L44, exerted through its content of air, may be a disadvantage in terms of distension and extension of the SB compared to its liquid counterparts. Still, L44 distributed well throughout the SB down to the terminal ileum and proximal colon in most patients. As the negative HU value after L44 administration is important for the diagnosis of lesions both within and outside the bowel, adequate distribution is pivotal. The hypothesis of low frequency of AEs when using L44 with its high content of air (44%) in water seems to be supported by the current study. In addition, the taste and smell of L44 seemed to be agreeable, whereas the easiness to swallow the foam and the foam’s consistency were deemed neutral, but lower than for Omnipaque®. L44 gave a lower degree of fullness sensation at the end of the oral preparation compared to the other regimens.

As the bowel wall-to-lumen interface is greatly increased using L44, it might be permissible to suppose that detection and diagnosis of unexpected cases of SB mucosal diseases, be it inflammatory bowel disease, polyps, and carcinoids, may be facilitated by a general use of L44 prior to most abdominal CT-examinations. An abdominal CT with L44 may in many ways resemble an MR enterography. This is tested in an ongoing study comparing L44 abdominal CT and conventional MR enterography in patients with known or suspected Crohn’s disease.

A previous study has suggested that using neutral oral contrast is superior to positive oral contrast for visualising the vasculature using maximum intensity projections [9]. This possibility also holds true for L44 compared to positive oral contrast. Example images are uploaded as Additional file 6.

Initial experiments by the current research group have shown that L44 in addition to CT can be used in MR enterography. If L44 can be used as the sole oral regime in departments, independent of imaging modality, working routines would become easier. The initial experiments of L44 in MR enterography are promising, and studies are planned to pursue the use of L44 in MR imaging, in situations where today other oral regimes are used.

Limitations of the study are primarily related to the study aim, *i.e.*, to assess the technical performance of L44. This means that any pathology found was not in focus for the scientific report. The study has therefore not proved that a higher wall to lumen attenuation results in better diagnostic performance although it might be expected from a theoretical point of view. The examinations were read in standard manner with normal clinical written reports by radiologists in charge. Second, there was no patient with explicit pathology in the SB. Hence, we were unable to demonstrate any benefit of the excellence of L44 in bowel wall-to-lumen contrast in patients with SB pathology. Third, it is too early to state that radiologists in general will appreciate images of the SB with dark lumen on CT. In addition, a limitation is the performed interim analysis as it was not defined in advance in the study protocol.

In conclusion, L44 is a novel negative oral contrast agent with a luminal HU-value of approximately -400 HU, creating a unique appearance and delineation of the SB on abdominal CT. The safety profile of L44 seems very good, and it was well accepted by the patients studied. The high bowel wall-to-lumen contrast of L44 encourages to pursue studies in patients with pathology in the abdomen and/or in the SB to study L44´s performance in comparison with current regimens.

## Supplementary Information

**Additional file 1: Fig. S1.** Flow-chart showing the accrual to the study, randomisation and imaging examinations.

**Additional file 2: Table S1-4.** Supplementary tables showing demographics and medical history of the patients studied.

**Additional file 3: Fig. S2.** Additional cine images showing Lumentin 44 in axial and coronal planes.

**Additional file 4: Fig. S3.** Additional cine images showing Omnipaque in axial and coronal planes.

**Additional file 5: Fig. S4.** Additional cine images showing MoviPrep in axial and coronal planes.

**Additional file 6: Fig. S5.** An image from the Lumentin 44 group visualising the vasculature using maximum intensity projection and corresponding image from the Omnipaque group showing the positive contrast to conceal much of the vasculature.

## Abbreviations

AEs: Adverse events
CT: Computed tomography
HU: Hounsfield units
L44: Lumentin 44
MR: Magnetic resonance
OCA: Oral contrast agent
PACS: Picture archiving and communication system
ROI: Region of interest
SB: Small bowel

## References

[1] ACR–SPR (2016) Practice parameter for the performance of computed tomography (CT) of the abdomen and computed tomography (CT) of the pelvis. https://www.acr.org/-/media/ACR/Files/Practice-Parameters/ct-abd-pel.pdf. Revised 2021

[2] Basile J, Kenny JF, Khodorkovsky B, et al (2018) Effects of eliminating routine use of oral contrast for computed tomography of the abdomen and pelvis: a pilot study. Clin Imaging 49:159–162. 10.1016/j.clinimag.2018.03.002 10.1016/j.clinimag.2018.03.002 29529452

[3] Taylor MB, Bromham NR, Arnold SE (2012). Carcinoma of unknown primary: key radiological issues from the recent National Institute for Health and Clinical Excellence guidelines. Br J Radiol.

[4] Pickhardt PJ (2020). Positive oral contrast material for abdominal CT: current clinical indications and areas of controversy. AJR Am J Roentgenol.

[5] Fork FT, Aabakken L (2007) Capsule enteroscopy and radiology of the small intestine. Eur Radiol 17:3103–3111. 10.1007/s00330-007-0718-766. 10.1007/s00330-007-0718-7 17876583

[6] Fork FT, Karlsson N, Kadhem S, Ohlsson B (2012). Small bowel enteroclyses with magnetic resonance imaging and computed tomography in patients with failed and uncertained passage of a patency capsule. BMC Med Imaging Feb.

[7] Megibow AJ, Babb JS, Hecht EM, et al (2006) Evaluation of bowel distention and bowel wall appearance by using neutral oral contrast agent for multi-detector row CT. Radiology 238:87–95. 10.1148/radiol.2381041985 10.1148/radiol.2381041985 16293806

[8] Paulsen SR, Huprich JE, Fletcher JG, et al (2006) CT enterography as a diagnostic tool in evaluating small bowel disorders: review of clinical experience with over 700 cases. Radiographics 26:641–657; discussion 657-662. 10.1148/rg.263055162 10.1148/rg.263055162 16702444

[9] Berther R, Patak MA, Eckhardt B, Erturk SM, Zollikofer CL (2008). Comparison of neutral oral contrast versus positive oral contrast medium in abdominal multidetector CT. Eur Radiol.

[10] Baldwin GN (1978). Computed tomography of the pancreas: negative contrast medium. Radiology.

[11] Raptopoulos V, Davis MA, Davidoff A, et al (1987) Fat-density oral contrast agent for abdominal CT. Radiology 164:653–656. 10.1148/radiology.164.3.3615862 10.1148/radiology.164.3.3615862 3615862

[12] Thompson SE, Raptopoulos V, Sheiman RL, McNicholas MM, Prassopoulos P (1999). Abdominal helical CT: milk as a low-attenuation oral contrast agent. Radiology.

[13] Ramsay DW, Markham DH, Morgan B, Rodgers PM, Liddicoat AJ (2001). The use of dilute Calogen as a fat density oral contrast medium in upper abdominal computed tomography, compared with the use of water and positive oral contrast media. Clin Radiol.

[14] Wei X, Zhu J, Gong H, Xu J, Xu Y (2011). A novel foam fluid negative contrast medium for clear visualization of the colon wall in CT imaging. Contrast Media Mol Imaging.

[15] Leander P, Adnerhill I, Böök O, Casal-Dujat L, Stathis G, Fork T (2021). A novel food based oral contrast agent with negative Hounsfield units for demarcation of small bowel loops on abdominal CT. Acta Radiol..

[16] Minordi LM, Vecchioli A, Mirk P, Bonomo L (2011) CT enterography with polyethylene glycol solution vs CT enteroclysis in small bowel disease. Br J Radiol 84:112–119. 10.1259/bjr/71649888 10.1259/bjr/71649888 PMC3473850 20959377

[17] Clason DL, Dormody TJ (1994) Analyzing data measured by individual Likert-type items. J Agric Educ 35:31–35

[18] Allen E, Seaman CA (2007). Likert scales and data analyses. Q Progr.

[19] Wang J, Nguyen AV, Farrokhpay S (2016). A critical review of the growth, drainage and collapse of foams. Adv Colloid Interface Sci.

[20] Hill C, Eastoe J (2017). Foams: from nature to industry. Adv Colloid Interface Sci.

