# Supplementary material for: A novel food-based negative oral contrast agent compared with two conventional oral contrast agents in abdominal CT: a three-arm parallel blinded randomised controlled single-centre trial
Source: Eur Radiol Exp. 2022 Apr 5;6:15. doi: 10.1186/s41747-022-00267-z (PMC8980139; doi:10.1186/s41747-022-00267-z)
Supplement: Supplementary file 1 — Additional file 1: Fig. S1. Flow-chart showing the accrual to the study, randomisation and imaging examinations. [file 41747_2022_267_MOESM1_ESM.pptx]

## Slide 1
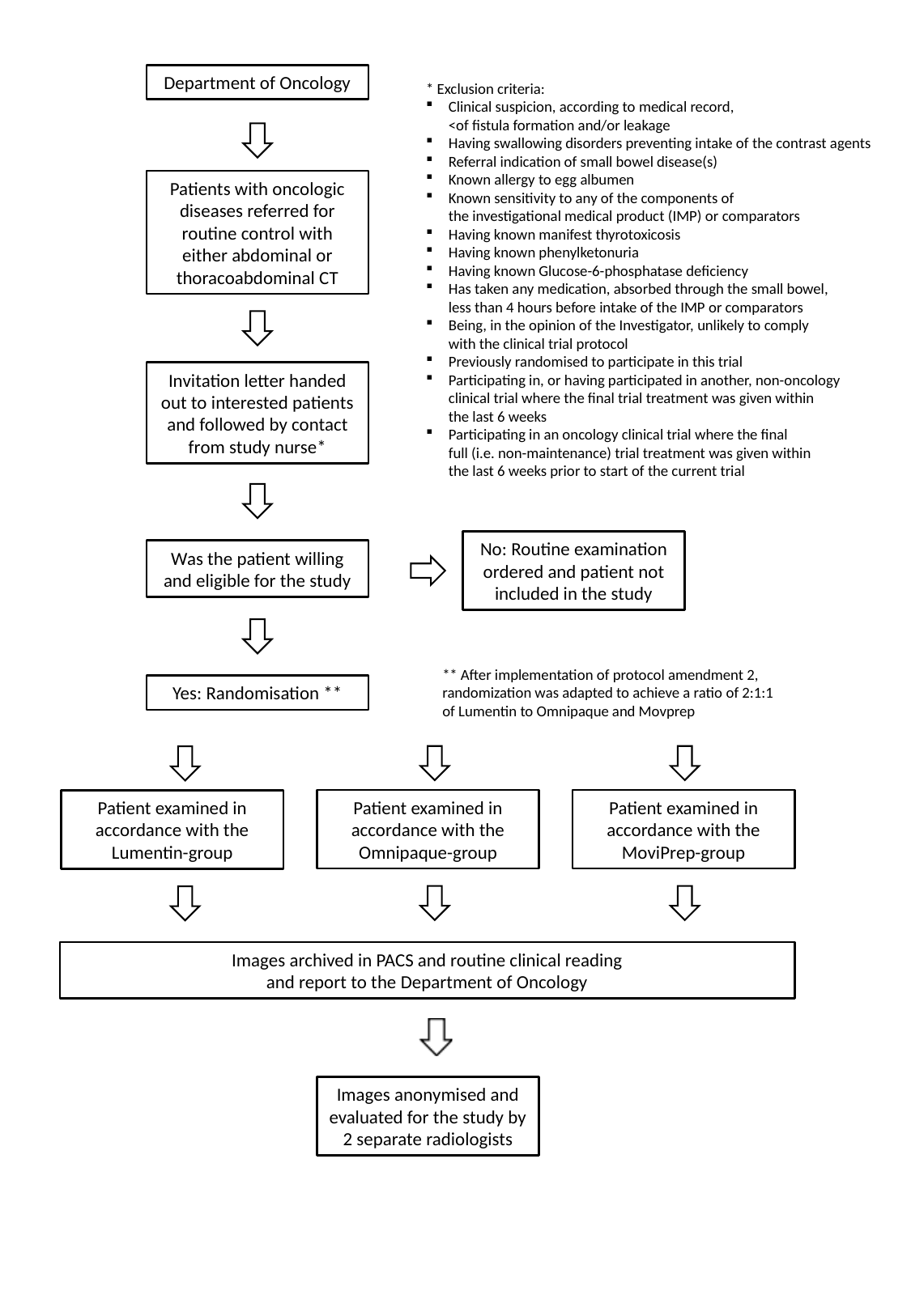

Department of Oncology
* Exclusion criteria:
Clinical suspicion, according to medical record,
<of fistula formation and/or leakage
Having swallowing disorders preventing intake of the contrast agents
Referral indication of small bowel disease(s)
Known allergy to egg albumen
Known sensitivity to any of the components of
the investigational medical product (IMP) or comparators
Having known manifest thyrotoxicosis
Having known phenylketonuria
Having known Glucose-6-phosphatase deficiency
Has taken any medication, absorbed through the small bowel,
less than 4 hours before intake of the IMP or comparators
Being, in the opinion of the Investigator, unlikely to comply
with the clinical trial protocol
Previously randomised to participate in this trial
Participating in, or having participated in another, non-oncology
clinical trial where the final trial treatment was given within
the last 6 weeks
Participating in an oncology clinical trial where the final
full (i.e. non-maintenance) trial treatment was given within
the last 6 weeks prior to start of the current trial
Patients with oncologic diseases referred for routine control with either abdominal or thoracoabdominal CT
Invitation letter handed out to interested patients and followed by contact from study nurse*
No: Routine examination ordered and patient not included in the study
Was the patient willing and eligible for the study
** After implementation of protocol amendment 2,
randomization was adapted to achieve a ratio of 2:1:1
of Lumentin to Omnipaque and Movprep
Yes: Randomisation **
Patient examined in accordance with the Omnipaque-group
Patient examined in accordance with the MoviPrep-group
Patient examined in accordance with the Lumentin-group
Images archived in PACS and routine clinical reading
and report to the Department of Oncology
Images anonymised and evaluated for the study by 2 separate radiologists
