# Supplementary material for: A novel food-based negative oral contrast agent compared with two conventional oral contrast agents in abdominal CT: a three-arm parallel blinded randomised controlled single-centre trial
Source: Eur Radiol Exp. 2022 Apr 5;6:15. doi: 10.1186/s41747-022-00267-z (PMC8980139; doi:10.1186/s41747-022-00267-z)
Supplement: Supplementary file 2 — Additional file 2: Table S1-4. Supplementary tables showing demographics and medical history of the patients studied. [file 41747_2022_267_MOESM2_ESM.docx]

**ELECTRONIC SUPPLEMENTARY MATERIAL**

**A novel food-based negative oral contrast agent compared with two conventional oral contrast agents in abdominal CT: a three-arm parallel blinded randomised controlled single-centre trial**

Supplementary tables showing demographics and medical history of the patients studied

Supplementary table 1

Demographic data by treatment groups. Figures denote numbers (N, n), percentage (%), Mean (SD), and Median (min; max)

| Variable | Total (N=43) | Lumentin 44 (N=17) | 10% Omnipaque (N=12) | Moviprep (N=14) |
| --- | --- | --- | --- | --- |
| Age (all) | 63.0 (15.4) 65.0  (20.0; 86.0) | 64.7 (16.2) 68.0  (32.0; 81.0) | 64.9 (14.1) 65.0  (39.0; 86.0) | 59.4 (15.8) 62.0  (20.0; 84.0) |
| Age group |  |  |  |  |
| 18-64 | 19 (44.2) | 6 (35.3) | 5 (41.7) | 8 (57.1) |
| 65-84 | 23 (53.5) | 11 (64.7) | 6 (50.0) | 6 (42.9) |
| 85- | 1 (2.3) | 0 (0.0) | 1 (8.3) | 0 (0.0) |
| Gender |  |  |  |  |
| Male | 18 (41.9) | 8 (47.1) | 4 (33.3) | 6 (42.9) |
| Female | 25 (58.1) | 9 (52.9) | 8 (66.7) | 8 (57.1) |
| Height (cm) | 170.0 (10.5) 168.0 (150.0; 195.0) | 168.9 (10.5) 170.0 (150.0; 185.0) | 168.0 (8.6) 168.0 (150.0; 182.0) | 172.9 (11.8) 171.0 (160.0; 195.0) |
| Weight (kg) | 78.3 (15.9) 77.0 (50.0; 130.0) | 84.9 (16.4) 84.0 (62.0; 130.0) | 71.8 (11.1) 72.5 (50.0; 87.0) | 75.9 (16.7) 72.5 (53.0; 115.0) |

Supplementary table 2

Medical history. Figures denote numbers (N, n), percentage (%)

|  | Lumentin 44  N = 17 | 10% Omnipaque  N = 12 | Moviprep  N = 14 |
| --- | --- | --- | --- |
| Medical history reported | 18 * | 11** (91.7) | 13** (92.9) |
|  |  |  |  |
| Cardiac disorder |  | 2 (16.6) |  |
| Endocrine disorder |  |  | 2 (14.2) |
| Gastrointestinal disorder |  |  | 1 (7.1) |
|  |  |  |  |
| Neoplasms | 18 (*) | 9 (75.0) | 10 (71.4) |
|  |  |  |  |
| Uro-genital | 6 (35,3) | 2 (16,7) | 3 (21,4) |
| Gastrointestinal | 7 (41.2) | 1 (8.3) | 3 (21.4) |
| Breast | 4 (23.6) | 5 (41.7) | 2 (14.2) |
| Miscellaneous | 1 (5.9) | 1 (8.3) | 2 (14.2) |

* 1 patient with two neoplasms

** 1 suspected neoplasm of unknown origin

Supplementary table 3

Subject disposition. Figures denote number (N, n) and (percentage)

| Variable | Total (N=45) | Lumentin 44 (N=19) | 10% Omnipaque (N=12) | Moviprep (N=14) |
| --- | --- | --- | --- | --- |
| Screened | 45 (100.0) | 19 (100.0) | 12 (100.0) | 14 (100.0) |
| Randomized | 45 (100.0) | 19 (100.0) | 12 (100.0) | 14 (100.0) |
| Completed | 41 (91.1) | 16 (84.2) | 12 (100.0) | 13 (92.9) |
|  |  |  |  |  |
| Safety set | 44 (97.8) | 18 (94.7) | 12 (100.0) | 14 (100.0) |
| Full analysis (FA) set | 43 (95.6) | 17 (89.5) | 12 (100.0) | 14 (100.0) |
| Per protocol (PP) set | 43 (95.6) | 17 (89.5) | 12 (100.0) | 14 (100.0) |

Supplementary table 4

Results by randomisation group for Body Mass Index (BMI), amount oral contrast (ml) and the ratio between the amount oral contrast and BMI.

Lumentin 44 (L44); 10% Omnipaque (OMNI)

| Group | Statistica | BMI | Oral contrast (mL) | Oral contrast / BMI |
| --- | --- | --- | --- | --- |
| Lumentin 44  N=17 | Mean (SD)  Median  (min; max) | 29.89 (5.90)  29.07  (21.91; 44.98) | 997.47 (43.64)  988  (937.00; 1109.00) | 34.42 (5.95)  34.11  (21.99; 46.06) |
| 10% Omnipaque  N=12 | Mean (SD)  Median  (min; max) | 25.43 (3.62)  24.41  (22.13; 34.41) | 1061 (34.62)  1059.50  (1000.00; 1144.00) | 42.42 (5.47)  42.99  (30.48; 51.48) |
| Moviprep  N=14 | Mean (SD)  Median  (min; max) | 25.18 (3.47)  25.34  (20.45; 31.22) | 988.64 (150.81)  1059.00  (633.00; 1137.00) | 39.61 (6.15)  40.94  (25.46; 49.09) |
| P-values (2-sided)  Wilcoxon rank sum test | L44 vs OMNI | 0.0214 | 0.0001 | 0.0019 |
|  | L44 vs MOV | 0.0119 | 0.4742 | 0.0166 |
