# Supplementary figures and images for: A novel food-based negative oral contrast agent compared with two conventional oral contrast agents in abdominal CT: a three-arm parallel blinded randomised controlled single-centre trial
Source: Eur Radiol Exp. 2022 Apr 5;6:15. doi: 10.1186/s41747-022-00267-z (PMC8980139; doi:10.1186/s41747-022-00267-z)

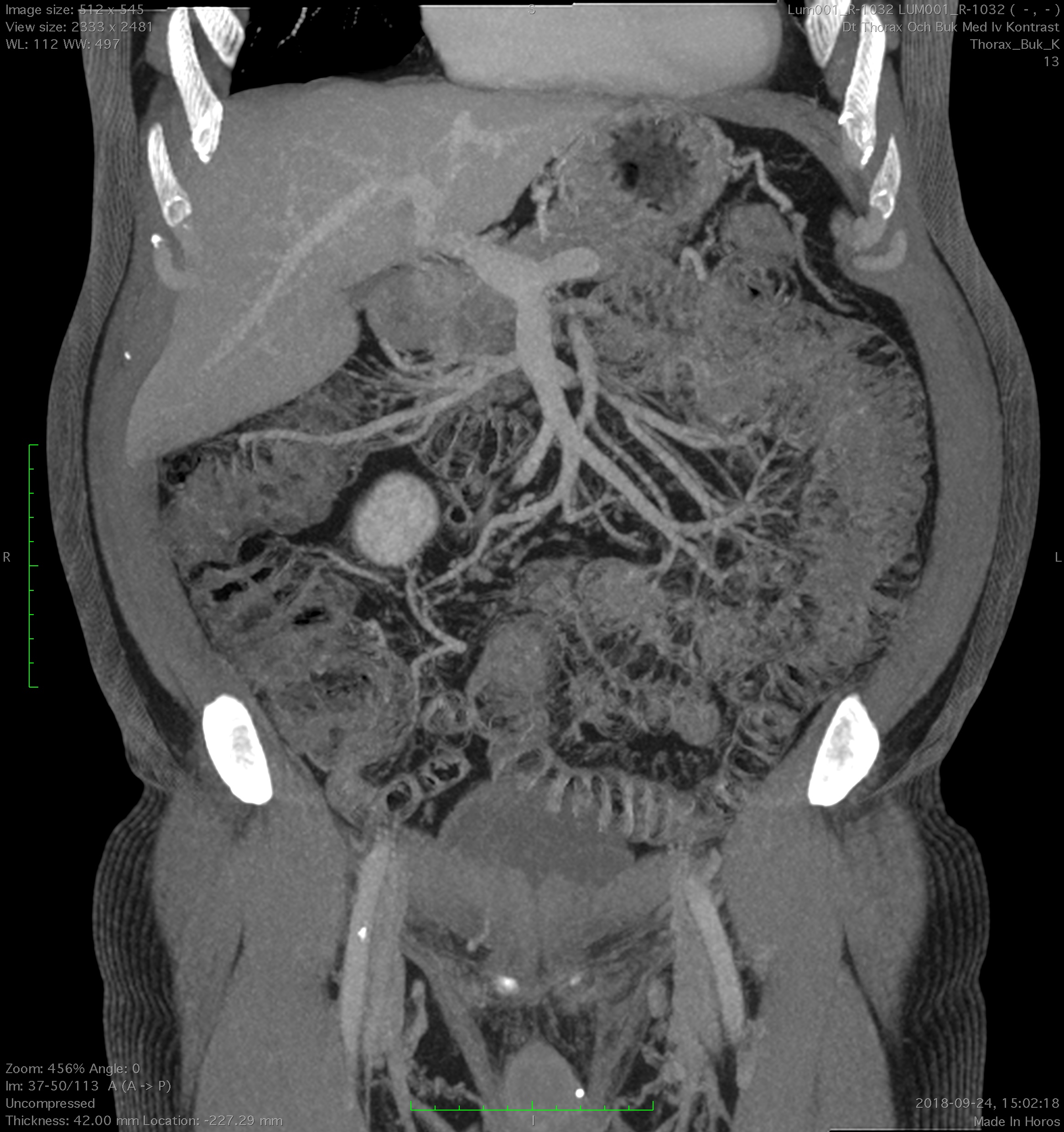

Supplement: Supplementary file 6 — Additional file 6: Fig. S5. An image from the Lumentin 44 group visualising the vasculature using maximum intensity projection and corresponding image from the Omnipaque group showing the positive contrast to conceal much of the vasculature. [file 41747_2022_267_MOESM6_ESM.zip › Additional file 6/EURE-D-21-00045_ESM_S5_Lumentin 44.jpg]

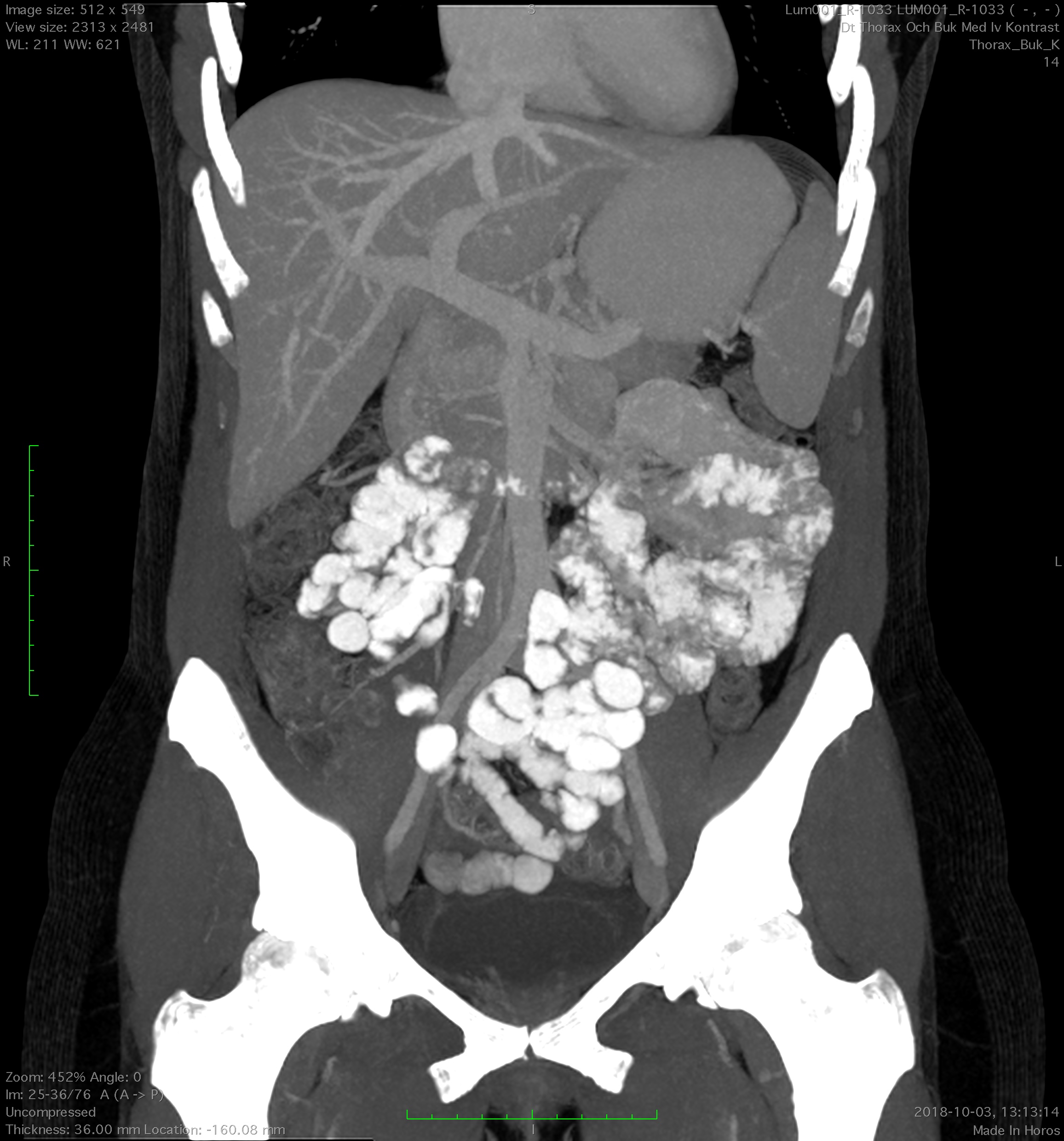

Supplement: Supplementary file 6 — Additional file 6: Fig. S5. An image from the Lumentin 44 group visualising the vasculature using maximum intensity projection and corresponding image from the Omnipaque group showing the positive contrast to conceal much of the vasculature. [file 41747_2022_267_MOESM6_ESM.zip › Additional file 6/EURE-D-21-00045_ESM_S5_Omnipaque Coronal.jpg]
